# Cell Cycle Arrest by Supraoptimal Temperature in the Alga *Chlamydomonas reinhardtii*

**DOI:** 10.3390/cells8101237

**Published:** 2019-10-11

**Authors:** Vilém Zachleder, Ivan Ivanov, Milada Vítová, Kateřina Bišová

**Affiliations:** 1 Laboratory of Cell Cycles of Algae, Centre Algatech, Institute of Microbiology of the Czech Academy of Sciences, 37981 Třeboň, Czech Republic; zachleder@alga.cz (V.Z.); ivanov@alga.cz (I.I.); vitova@alga.cz (M.V.); 2 Faculty of Science, University of South Bohemia, Branišovská 1700, 37005 České Budějovice, Czech Republic

**Keywords:** cell cycle arrest, cell size, *Chlamydomonas reinhardtii*, cyclin-dependent kinase, DNA replication, synchronized cultures, supraoptimal temperature, starch accumulation

## Abstract

Temperature is one of the key factors affecting growth and division of algal cells. High temperature inhibits the cell cycle in *Chlamydomonas reinhardtii*. At 39 °C, nuclear and cellular divisions in synchronized cultures were blocked completely, while DNA replication was partly affected. In contrast, growth (cell volume, dry matter, total protein, and RNA) remained unaffected, and starch accumulated at very high levels. The cell cycle arrest could be removed by transfer to 30 °C, but a full recovery occurred only in cultures cultivated up to 14 h at 39 °C. Thereafter, individual cell cycle processes began to be affected in sequence; daughter cell release, cell division, and DNA replication. Cell cycle arrest was accompanied by high mitotic cyclin-dependent kinase activity that decreased after completion of nuclear and cellular division following transfer to 30 °C. Cell cycle arrest was, therefore, not caused by a lack of cyclin-dependent kinase activity but rather a blockage in downstream processes.

## 1. Introduction

Light and temperature are two key factors that affect growth and development of plants, including green algae. For each organism, there are three temperature ranges with distinct effects on cell physiology. Firstly, the optimum growth temperature is where growth rate reaches a maximum. Secondly, the range below and above the optimum is where growth and cell division are possible, yet at lower rates. The final range is temperatures too low or too high to allow for cell division, cell growth, or cell survival. Within the physiological range surrounding the optimum, a 10 °C increase in temperature will increase metabolic rate two-fold. Increasing temperature will thus speed up growth as well as shorten individual phases of the cell cycle and its total duration, as shown in different algae such as *Chlorella ellipsoidea* [1], *Chlamydomonas reinhardtii* [2], *Chlamydomonas eugametos* [3], and *Desmodesmus quadricauda* [4]. Individual metabolic processes are differentially sensitive to temperature, which contributes to distinct cell responses within a temperature range. Cell division and duration of the cell cycle seem to be more sensitive to temperature than growth. Cultures of *C. reinhardtii* grown at increasing temperatures will, at first, increase growth rates and shorten the cell cycle, as predicted by the two-fold increase in metabolic rate [2]. With a further temperature increase between 28 and 37 °C, duration of the cell cycle will be prolonged, while the growth rates will remain comparable. Since a temperature increase of 10 °C should double the growth rate, maintaining similar growth rates between the two temperatures implies that the growth rate effectively decreases in contrast to predictions. Thus, although the cells are not yet visibly stressed, such temperatures are physiologically supraoptimal. Would a further increase in temperature lead to cell division arrest? When optimizing growth conditions for synchronized cultures of *C. reinhardtii,* Lien and Knutsen noted that at 1 °C above the optimal growth temperature, some cells started to exhibit inhibited cell division [5]. But such effects might be so subtle that they can only be detected in synchronized cultures when the entire culture is of a similar age. In distantly related alga, *Chlorella vulgaris,* an increase in temperature of 6–7 °C above the growth optima arrested nuclear and cellular divisions, but not DNA replication, and the effect on growth was negligible [6]. Cell cycle arrest thus seems to be one of the first physiological processes affected by even small increases in temperature above the optimum, but the nature of the arrest remains unknown. It is unclear if the arrest is caused by an effect on cell cycle regulatory protein activities (such as cyclin-dependent kinases) or by an effect on downstream cell cycle events.

**Figure 1:**
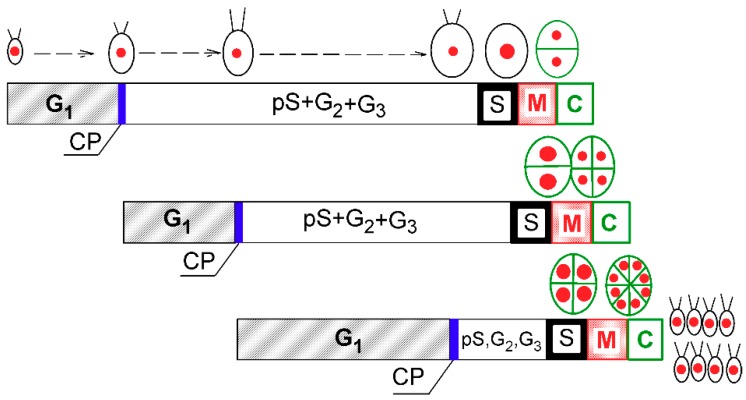
Schematic diagram of growth and the cell cycle in a single cell of *Chlamydomonas reinhardtii*. The schematic pictures reflect increasing cell size. The red full circles inside the cells illustrate the size and number of nuclei. Larger red circles indicate a doubling of DNA. Green ellipsoids and lines represent protoplast division. Diagram shows a model of cell cycle progression in *C. reinhardtii* dividing by multiple fission into 8 daughter cells. Three bars indicate three overlapping growth and reproductive sequences terminated by division into 2, 4, and 8 daughter cells, respectively. Precommitment period (G1): the period until threshold critical cell size for commitment to divide (CP) is reached and CP is attained. Postcommitment period consists of pS—the prereplication phase between the CP attainment and the beginning of DNA replication. The processes required for initiation of DNA replication are assumed to happen during this phase. S: DNA replication takes place. G2: the phase between the termination of DNA replication and the start of mitosis (M). Processes leading to the initiation of mitosis are assumed to take place during this phase. G3: the phase separating mitosis from cellular division, which is clearly visible in some algae dividing by multiple fission. The processes leading to cellular division are assumed to take place during this phase. C: the phase during which cell cleavage (protoplast fission) and daughter cell formation occurs. For *C. reinhardtii*, it is typical that all “gap” phases are combined into one prolonged phase. The distances between individual cell cycle phases correspond to the cell cycle progression in a real culture (Figure 2). Modified after Zachleder et al. [13], Bišová and Zachleder [14], and Zachleder et al. [15].

*C. reinhardtii* is a model species that divides by multiple fission. Its cell cycle can be modeled as a series of overlapping reproductive sequences, each of them consisting of cell cycle entry at commitment point (CP) that switches on DNA replication (S phase), nuclear division (M phase), and cell division (C) (Figure 1) [5,7,8,9,10]. During growth in G1 phase, cells attain their first CP, which would lead to completion of a single reproductive sequence (i.e., division into two daughter cells). At sufficiently fast growth rates, they may also attain consecutive CPs (*n*), each of which will eventually lead to completion of one reproductive sequence [11,12], independent of further energy supply (i.e., even in the dark). The mother cell can, therefore, divide into 2, 4, 8, or 16 (2*^n^*) cells [5].

In single algal cells (and in synchronized cultures that mimic them), growth is manifested by an increase in cell size, RNA, protein, and starch (and other energy components) per cell, which leads to the accumulation of dry matter and an increase in absorbance (A_750_) of the culture. Growth is a prerequisite for cell division, as the attainment of each CP depends on reaching a critical cell size [10,16], which involves a doubling of cell size, cell mass, RNA, and protein per cell [2,11,12,15,17]. Growth is, therefore, tightly connected with the cell cycle so the number of reproductive sequences and released daughter cells are dictated by cell size [10,16]. Moreover, although there might be growth in the absence of cell division, division in the absence of growth leads to decreasing cell size and is, thus, limited to specific cases. Temperature affects growth as well as the entire cellular metabolism. Thus, it affects the duration of the growth phase prior to attainment of CP, the precommitment period, as well as the postcommitment period, where, under most conditions, growth runs concurrently with reproductive sequences [2,3,4]. This is in contrast to light that only affects growth and the duration of the precommitment period, but the postcommitment period remains constant [3,17]. Thus, the effect of temperature on the cell cycle is quite complex, even within the physiological range.

In plants, including algae, the cell cycle is regulated by two types of cyclin-dependent kinases (CDKs), CDKA and plant-specific CDKB. *C. reinhardtii* CDKA [18] and CDKB homologues are encoded by single genes [19] and have nonoverlapping functions [20]. CDKA promotes entry into cell division at CP and is also required to initiate the first DNA replication [20]. CDKB is the specific mitotic kinase that is required for spindle formation, nuclear division, and subsequent rounds of S phase, but not for cytokinesis [20]. Only CDKB is essential, whilst the null mutant of CDKA prolongs growth and delays cell division [21].

In the present paper, we describe the effect of supraoptimal temperature on cell cycle arrest and recovery in synchronized cultures of *C. reinhardtii*. We show that a supraoptimal temperature inhibited cell reproduction and the expenditure of starch, while normal growth continued, leading to the formation of giant cells with elevated levels of starch. Cell cycle arrest comprised a block in nuclear and cellular division, while DNA replication was less affected. Arrest was accompanied by high mitotic kinase activities that declined following nuclear and cellular division/s. Cell cycle arrest was, therefore, not caused by a lack of cyclin-dependent kinase activity but rather a blockage in downstream processes.

## 2. Materials and Methods

### 2.1. Organism and Culture

The unicellular alga *Chlamydomonas reinhardtii* wild-type 21gr (CC-1690) was obtained from the Chlamydomonas Resource Center at the University of Minnesota (St. Paul, MN, USA). The cultures were grown on high salt medium (HS) as described by Sueoka [22] with a doubled concentration of Ca^2+^ ions and a tenfold increase in Mg^2+^ ions. Trace elements (1 mL per 1 L of medium) as described by Zachleder and Šetlík [23] were used instead of Hutner’s trace elements. For routine subculturing, the cultures were streaked every three weeks onto modified high salt medium solidified by agar and grown at an incident light intensity of 100 µmol m^−2^ s^−1^ of photosynthetically active radiation.

### 2.2. Synchronization Procedure

For synchronization, 300 mL of liquid HS medium was inoculated directly from plates, and the cultures were placed in glass cylinders (inner diameter 30 mm, height 500 mm) at 30 °C and “aerated” with a mixture of air and CO_2_ (2%, v/v) at a flow rate of 15 L h^−1^. The cylinders were illuminated from one side by a panel of dimmable fluorescent lamps (OSRAM DULUX L55W/950 Daylight, Milano, Italy) with light intensity adjusted to an incident light intensity of 500 µmol m^−2^ s^−1^ of photosynthetically active radiation at the surface of the cylinders. Synchronization was carried out by 13/11 h alternating light/dark (L/D) periods, as was described previously [24].

Suspensions of synchronous cells were diluted to a maximum concentration between 1.5 × 10^6^ and 2 × 10^6^ cells mL^−1^ and cultivated in rectangular plate-parallel vessels (440 × 245 × 23 mm, volume 2200 mL) at the same incident light conditions as used for synchronization. The flow rate of the aeration mix was 60 L h^−1^. Culture vessels were immersed in water baths kept at constant temperatures of either 30 or 39 °C. Each experiment was carried out in triplicate. Individual processes of the cell cycle were performed at the same time with midpoints varying by a maximum of one hour. Assessments of CP attainment, cell division, cell size, and cell number were carried out as described by Hlavová et al. [24]. Dry matter was determined according to Brányiková et al. [25].

### 2.3. Determination of Total DNA, RNA, Protein, and Starch

Total nucleic acid was extracted and analyzed according to Wanka [26], as modified by Lukavský et al. [27]. For DNA content, the light-activated reaction of diphenylamine with hydrolyzed DNA was used, as described by Decallonne and Weyns [28] with modifications of Zachleder [29]. The sediment remaining after nucleic acid extraction was used for protein determination after Lowry et al. [30]. Starch content was determined by the anthrone method [31] as modified by Brányiková et al. [25]. Two technical replicates were used for each analysis. Variations between duplicates did not exceed 5% of the mean.

### 2.4. Activity of Cyclin-Dependent Kinases

Protein lysates were prepared as described by Hlavová et al. [32]. They were directly assayed or affinity-purified by CrCKS1 beads as described by Bisova et al. [19] with modifications as described by Hlavová et al. [32]. Histone H1 kinase activity was assayed as previously described [33] in a final volume of 10 µL with either 7 µL of clear whole cell lysate or the CrCKS1 beads fraction corresponding to 20 µL of whole cell lysate. The reactions were initiated by adding the master mix to a final composition of 20 mM HEPES, pH 7.5, 15 mM MgCl_2_, 5 mM EGTA, 1 mM DTT, 0.1 mM ATP, 0.2% (*w*/*v*) histone (Sigma H5505) and 0.370 MBq [γ ^32^P] ATP. All the chemicals were purchased from Sigma-Aldrich (Prague, Czech Republic).

Proteins were separated on 15% SDS-PAGE gels [34]. Phosphorylated histone bands were visualized by autoradiography, analyzed using a Phosphoimager (Storm 860, Molecular Dynamics, GE Healthcare, Prague, Czech Republic), and quantified using Image Studio Lite software (LI-COR Biosciences, v. 5.2, Lincoln, NE, USA) as described by Zachleder et al. [4].

## 3. Results

### 3.1. Cell Cycle Progression Differs between 30 °C and 39 °C, but Growth Remains Unaffected

**Figure 2:**
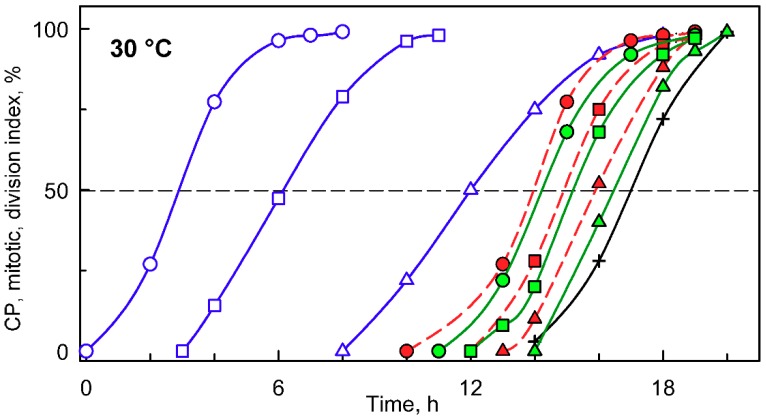
Graphical representation of the cell cycle progression in synchronized cultures of the *Chlamydomonas reinhardtii* grown at 30 °C at an incident light intensity of 500 μmol m^−2^ s^−1^. Time courses of individual commitment point/s, nuclear division/s, protoplast fission/s, and daughter cell release in synchronized cultures of *C. reinhardtii*. Blue curves (CP indices): cumulative percentage of cells, in a given population (100%), that attained the commitment point (CP) for the first (circles), second (squares), and third (triangles) reproductive sequence. Red curves (mitotic indices): cumulative percentage of cells, in a given population (100%), that divided their nuclei into 2 (circles), 4 (squares), and 8 (triangles). Green curves (division/protoplast fission indices): cumulative percentage of cells, in a given population (100%), that divided their protoplasts into 2 (circles), 4 (squares), and 8 (triangles). Black curve, crosses: cumulative percentage of cells that released daughter cells. Black dashed line at 50% depicts midpoint of each event in a population of cells.

When a synchronized culture was grown at 30 °C, it sequentially attained three CPs, with midpoints at approximately 3, 6, and 12 h after the onset of light (Figure 2, blue circles, squares and triangles, respectively). Each of the CPs switched on one reproductive sequence, leading to three rounds of nuclear division (Figure 2, red circles, squares and triangles, respectively) in a clustered pattern with midpoints within a short time interval of three hours between 13 and 16 h. Each of the nuclear divisions was immediately followed by protoplast fission (Figure 2, green circles, squares and triangles respectively), so no multinuclear intermediates occurred in the cells. The divided protoplasts remained attached within the mother cell wall until the freely moving flagellated daughter cells were formed and released with a midpoint at 17 h (for a schematic illustration, see Figure 1).

**Figure 3:**
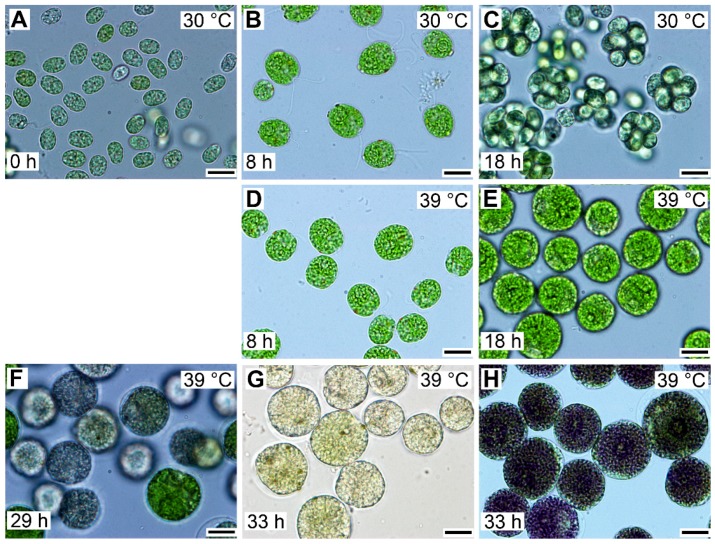
Photomicrographs of cells in synchronized cultures of *Chlamydomonas reinhardtii* grown in incident light 500 μmol m^−2^ s^−1^ at 30 °C (**A**–**C**) or 39 °C (**D**–**H**). The cultivation temperature and the cell age in hours are indicated in the pictures. The control cells, grown at 30 °C, divided at 18 h and thereafter entered a new cell cycle sequence similar to the one depicted in panels **A**–**C**. In contrast, the cells grown at 39 °C did not divide even after prolonged cultivation. They attained a maximum size after about 29 h of growth and started to lose chlorophyll (**F**). After 33 h, the majority of the cells lost chlorophyll and accumulated enormous amounts of starch in the form of grains (**G**), which can be visualized by staining with Lugol solution (**H**). Cultures at both 30 and 39 °C started from the same initial culture, so the cells depicted in panel A for 30 °C reflect also the cells present at 0 h at 39 °C. Bar = 10 µm.

**Figure 4:**
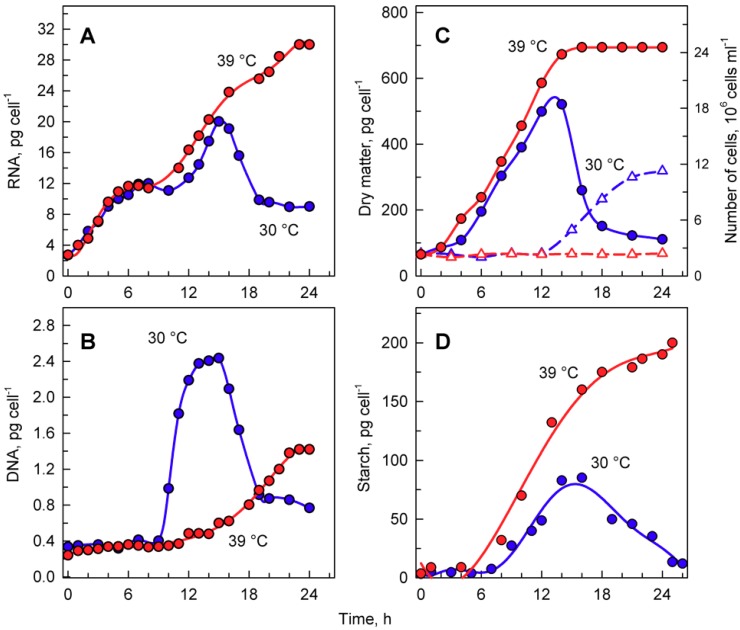
The course of RNA synthesis (**A**), DNA replications (**B**), changes in the number of cells and concentration of dry matter (**C**), and total starch (**D)** in the synchronized culture of *Chlamydomonas reinhardtii* grown under continuous illumination of incident light intensity 500 μmol m^−2^ s^−1^ at 30 °C (blue lines and symbols) or 39 °C (red lines and symbols).

Entry into the cell cycle was preceded by growth to a critical cell size. Microscopically, cells grown at 30 °C started as flagellated daughter cells (Figure 3A) that increased in size (Figure 3B) until they underwent cell division and gave rise to the next generation of daughter cells (Figure 3C), which could then undergo a similar sequence, provided that the growth conditions did not change. To study the effect of temperature on the cell cycle, we determined the lowest supraoptimal temperature that caused cell cycle arrest but did not affect growth. The heat stress temperature (42 °C) was not appropriate because this not only caused cell cycle arrest but also affected growth and cellular metabolism [35]. For *Chlamydomonas,* the optimal growth temperature was 35 °C, and at temperatures above 36 °C, protoplast fission and daughter cell release were inhibited [5]. Temperatures between 36 and 42 °C were, therefore, tested for their potential to cause cell cycle arrest. The lowest effective temperature was 39 °C, when protoplast division and daughter cell release were inhibited in 95%–100% of the population. In contrast, at 39 °C, comparing cell size and RNA (Figure 3 and Figure 4), cells grew similarly to controls. This was true until cells at 30 °C started to divide. Thereafter, the cells at 39 °C continued to grow for at least another 10 h, to more than double the size (1409.7 ± 50.7) of cells grown at 30 °C (640.3 ± 43.7) (Figure 3). These large cells then became pale due to decreased chlorophyll content and ceased growth (Figure 3F,G), but they retained a high content of starch (Figure 3G,H). The cells grown at 39 °C did not divide for the entire duration of the experiment (Figure 3G), corresponding to a duration of almost two control cell cycles.

The total RNA content (Figure 4A) doubled several times in cells grown at 30 °C, reached a maximum just before cellular division, and then decreased once the cells had divided (Figure 4A). DNA replication, as the first reproductive event, started by the 9th to 10th hour of the cell cycle and was completed by the 14th hour. During this time, DNA multiplied about 8-fold, corresponding to the production of 8 daughters per mother cell, and decreased again with cell division (Figure 4B). Cells grown at 39 °C accumulated RNA with similar kinetics to those of the control culture at 30 °C. They continued to grow and increased total RNA even at the time corresponding to division in the control cells, reaching about a 1.6-fold higher content of RNA compared to the maximum attained at 30 °C (Figure 4A). No division occurred at 39 °C. DNA replication at 39 °C started with about a 6 h delay, but it only increased about 3- to 4-fold compared to initial values and was, thus, more than 2-fold lower than the maximum seen in control cultures grown at 30 °C (Figure 4B).

### 3.2. Starch Reserves Accumulate and are Not Consumed at 39 °C

Growth at 30 and 39 °C was comparable with changes in total RNA content (Figure 4A) as well as for increases in dry matter (Figure 4C). The two cultures had virtually the same kinetics of dry matter increase up to the time when cellular divisions started in the control cultures (Figure 4C). In the control cultures, cell division led to a drop in dry matter per cell (Figure 4C), whilst the culture at 39 °C continued to increase dry matter for at least two more hours. 

Microscopic analysis indicated that cells grown at 39 °C accumulated starch (Figure 3G,H). In *C. reinhardtii*, starch is used as a primary storage molecule and serves as the energy supply for cell reproduction as well as dark metabolism. In the culture grown at 30 °C, starch steadily increased up to about 10-times the initial value. About the time when the cells divided, the starch content plateaued and then decreased, probably because it was used as a source of energy and carbon for cell reproduction (Figure 4D, blue circles). The culture grown at 39 °C accumulated starch much faster than at 30 °C (Figure 4D, red circles). The final total starch content per cell at 39 °C was more than 2-fold higher than that of the maximum reached at 30 °C (Figure 4D). The rate of accumulation of starch at 39 °C (Figure 4D) was much higher than that of other growth processes (total RNA, dry matter) at the same temperature (Figure 4A,C).

### 3.3. Cells Transferred from 39 °C to 30 °C Recover from Cell Cycle Arrest

**Figure 5:**
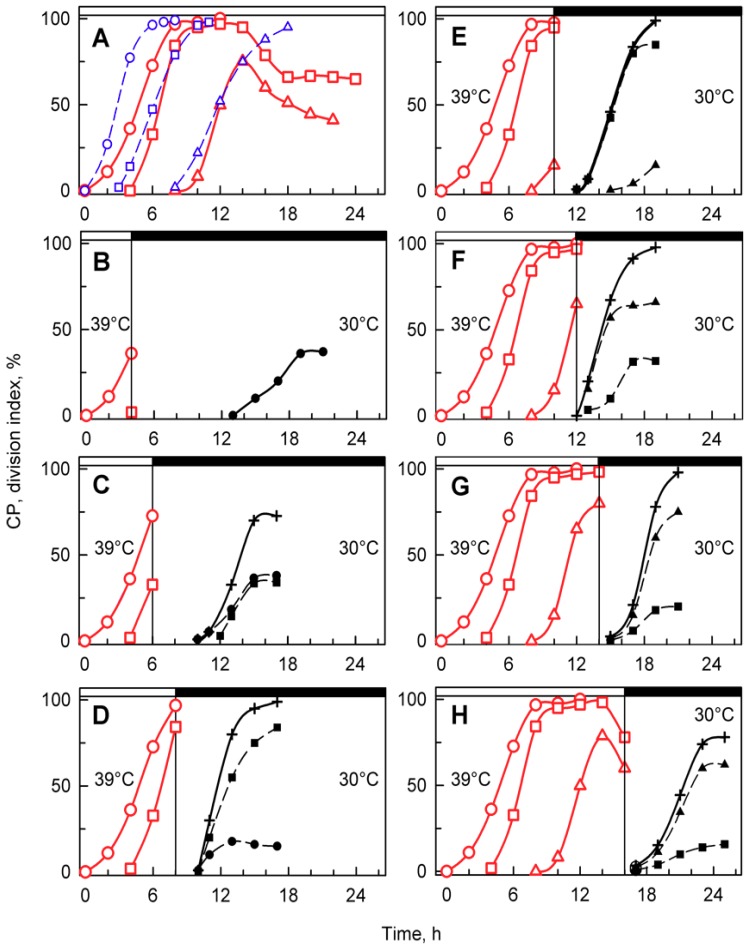
Time courses of individual CPs and cellular divisions in synchronized populations of *Chlamydomonas reinhardtii*. Panel **A**, the cells were grown in light at 30 °C (blue lines) or 39 °C (red lines); panels **B**–**H**, the cells were grown at 39 °C and then placed in the dark after 4, 6, 8, 10, 12, 14, and 16 h, respectively, and kept at 30 °C. No cell divisions occurred at 39 °C (**A**). Blue lines (CP indices): cumulative percentage of cells in a given population (100%) grown at 30 °C that attained commitment points (CPs) for the first (circles), second (squares), and third (triangles) reproductive sequences. Red lines (CP indices): the cells were grown at 39 °C and transferred bihourly to the dark at 30 °C. The cumulative percentage of 2, 4, and 8 daughter cells released in the dark in a given population (100%) is plotted as the attainment of the commitment points for the first (circles), second (squares), and third (triangles) reproductive sequences. Black dashed lines (division indices): cumulative percentage of cells that completed the first (full circles), second (full squares), and third (full triangles) cellular divisions and released 2, 4, or 8 daughter cells in a given population (100%). Black full lines, crosses: cumulative percentage of all cells in a given population (100%) that released daughter cells at 30 °C in dark. Times of transfer into darkness at 30 °C are indicated by vertical lines and by dark boxes above the graphs.

The effects of the supraoptimal temperature, 39 °C, seemed to be at least two-fold. Firstly, it induced cell cycle arrest, and secondly, in the absence of cell division, it caused a rapid accumulation of starch at rates exceeding synthesis of other metabolites. However, is the effect reversible? Cultures grown at 39 °C were transferred to the dark at 30 °C, where growth was inhibited due to the absence of an energy source, but the cell cycle continued. Control cultures grown at 30 °C attained 3 consecutive CPs, leading to initiation of the reproductive sequence for division into 2, 4, and 8 daughter cells (Figure 5A, blue lines), and thereafter divided into 8 daughter cells with a midpoint at the 17th hour (Figure 2).

Synchronized cells cultivated at 39 °C grew similarly to their 30 °C controls (Figure 3 and Figure 4), but nuclear division and protoplast fission were inhibited, and daughter cells were not formed (Figure 3) even when put into darkness at 39 °C. This demonstrated that cell cycle arrest was dependent solely on temperature and not on a combination of light and temperature stress. Because of the absence of cell division in dark at 39 °C, to assess attaining of CP, cells growing at 39 °C were transferred to darkness at 30 °C (Figure 5A, red curves). At this temperature, the cells completed nuclear and cellular divisions and produced daughter cells, suggesting that during the relatively undisturbed growth at 39 °C, CPs were, indeed, attained, but the corresponding reproductive processes were blocked. The attainment of CPs at 39 °C was similar to that at 30 °C, with the first CP being delayed by about two hours at 39 °C, and the proportion of the population attaining the third CP for division into eight daughter cells was about 25% lower than controls (Figure 5A). After 16 or more hours of cultivation at 39 °C, the cells were no longer capable of complete recovery from the effect of high temperature, and the number of committed cells started to decrease (Figure 5A). The relationship between growth and CP attainment was maintained, as the proportion of dividing daughter cells produced in the dark increased with the prolongation of growth at 39 °C. Consequently, the number of daughter cells formed in the population that was transferred to darkness at 30 °C increased (Figure 5B–G) until the inhibitory effect of prolonged cultivation at 39 °C was manifested from the 16th hour (Figure 5H). Daughter cell release occurred at about the same time in cultures darkened at the 6th, 8th, 10th, and 12th hours (Figure 5C–F). It was slightly delayed in transfers after the 4th and 14th hours (Figure 5B,G) and delayed most in cultures transferred after the 16th hour. The delay in daughter cell release in cultures transferred at the 14th and 16th hour was most probably dictated by the fact that the entire reproductive sequence, including mitosis, protoplast fission, and daughter cell release, could have proceeded only after transfer to 30 °C.

**Figure 6:**
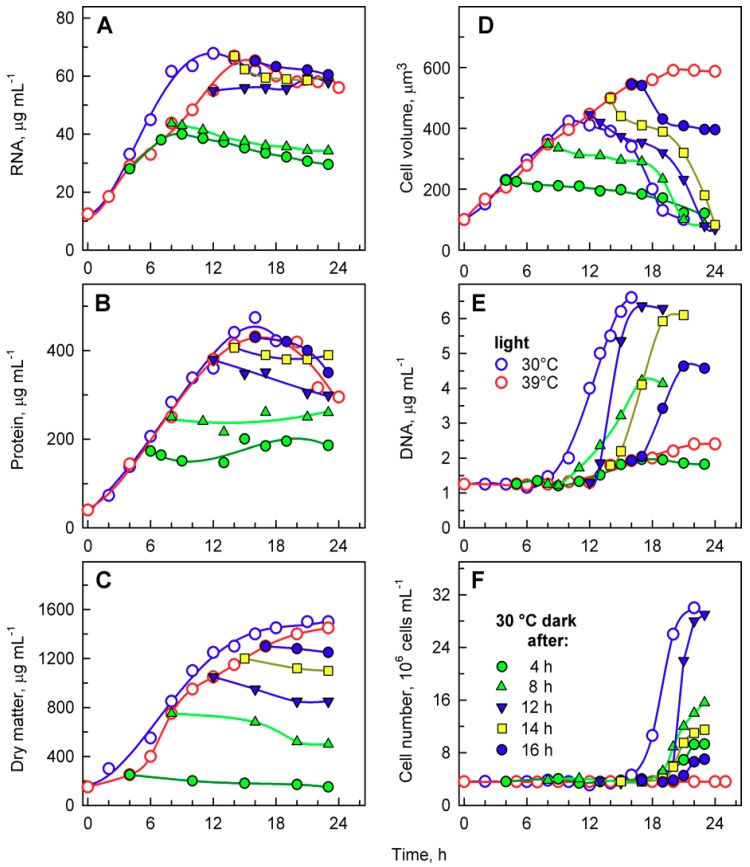
Changes in total RNA (**A**), total protein (**B**), dry matter (**C**), mean cell volume (**D**), DNA (**E**), and number of cells (**F**) in synchronized populations of *Chlamydomonas reinhardtii* grown at 30 °C (blue open circles), 39 °C (red open circles), and transferred from 39 °C into the dark at 30 °C at the 4th hour (green solid circles), 8th hour (green solid triangles), 12th hour (blue solid converted triangles), 14th hour (yellow solid squares), and 16th hour (blue solid circles), respectively.

A more detailed analysis of the dark 30 °C recovery from growth at 39 °C confirmed that growth ceased once the cells were put into darkness. This is clear from changes in total RNA and protein as well as the amount of dry matter that remained constant after the dark transfer or was slightly reduced, probably due to using some reserves for dark metabolism (Figure 6A–C). Based on changes in mean cell volume, cells transferred to darkness at 30 °C, after 4 and 8 h at 39 °C, started to release daughter cells with a slight delay compared to control cells grown at 30 °C (Figure 6D); this was also confirmed by the increase in cell number (Figure 6F). Daughter cell release was delayed by 4–6 h in cultures transferred at 12 and 14 h (Figure 6D,F). Cells transferred after 16 h were not only slightly delayed in daughter cell release, as shown by changes in mean cell volume (Figure 6D), but the number of daughter cells released (2.6-fold increase) was lower than the number expected from direct counting of cell divisions (3.8-fold increase; compare Figure 5H and Figure 6F). This was caused by some of the divided daughter cells not hatching and staying connected in clumps.

DNA replication increased with the length of incubation in light at 39 °C, from 4 to 14 h (Figure 6F), but started to decrease from 16 h (Figure 6F). The timing of DNA replication reflected the timing of cell division (compare Figure 5 and Figure 6E), as is typical for *C. reinhardtii*. DNA replication was delayed by about 2 h in cultures transferred after 8 and 12 h (Figure 6E). The delay increased to 6 h for cultures transferred after 14 h, and to 8 h for cultures transferred after 16 h (Figure 6E). The delay suggests that DNA replication was inhibited by the supraoptimal temperature treatment and could fully recover only after transfer to 30 °C. The number of daughter cells released also increased with the length of incubation in light at 39 °C, from 4 to 12 h (Figure 6F), and decreased thereafter (Figure 6F). These data (Figure 6E,F) confirmed results from direct microscopy counts of the number of daughter cells per mother cell (Figure 5) and further strengthened the idea of an inhibitory effect of prolonged incubation at supraoptimal temperatures. The extent of the effect was manifested in descending order, from daughter cell release, number of daughter cells formed per mother cell, and extent of DNA replication, so that DNA replication was the last affected, and daughter cell release was the first to be altered by the supraoptimal temperature.

### 3.4. CDK Activity Changes Upon a Shift to Darkness at 30 °C

A supraoptimal temperature was shown to block the cell cycle, particularly nuclear and cellular divisions, while DNA replication proceeded, albeit later and to a lesser extent. Some of the cell cycle regulatory machinery must, therefore, be active to switch on DNA replication. However, it is not clear whether the limiting factor blocking the cell cycle is related to the activity of key cell cycle regulators, cyclin-dependent kinases (CDKs), or whether activity was not affected and the cell cycle was blocked further downstream. In *C. reinhardtii* there are two cell cycle regulatory CDKs, CDKA and CDKB [19,20]. Temperature-sensitive mutants of both were isolated [20], and their cell cycle regulatory functions were analyzed in detail [21,36]. Unfortunately, because of the temperature-sensitivity of the mutants, they could not be used in our experiments. Instead, we adopted a more general analysis of CDK activity in two types of protein mixtures containing different proportions of CDK complexes. Firstly, we analyzed CDK activity in whole cell lysates. Such unpurified extracts contained all the CDK complexes present at the same time in the cells; activity was related to attaining CP as well as to the completion of nuclear and cellular divisions [37]. Specific mitotic CDK complexes were purified through their affinity to bind to CrCKS1 protein. Activity bound to CrCKS1 protein beads corresponded almost exclusively to the completion of nuclear and cellular divisions [19] and encompassed activities of both CDKA and CDKB complexes [20]. Thus, although both types of protein extracts contained CDKA as well as CDKB complexes, the proportions of the two complexes in the mixture, as well as their activities, might differ because of the isolation procedure.

**Figure 7:**
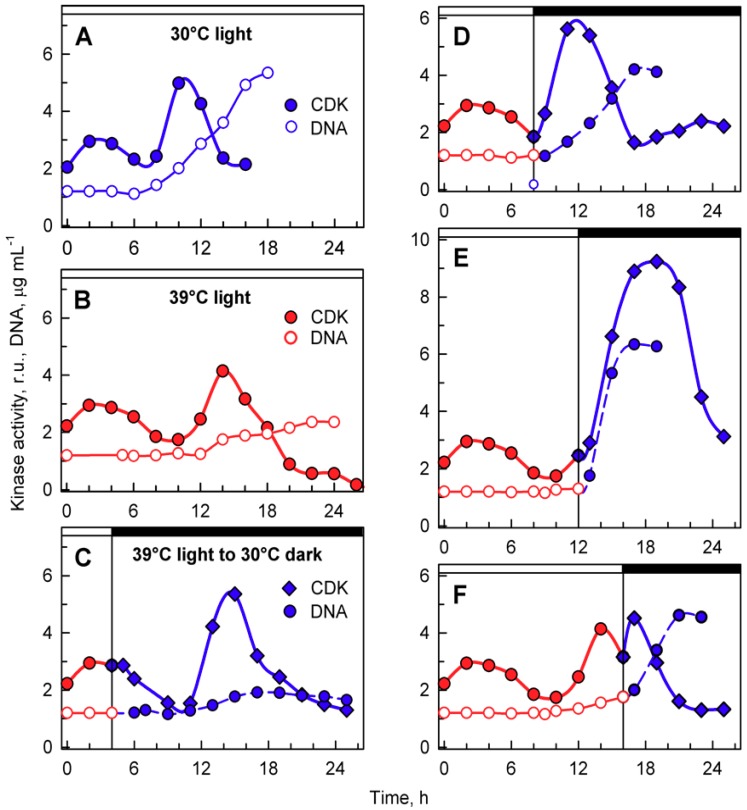
Activity of cyclin-dependent kinase (CDK) in whole-cell lysates prepared from synchronized cultures of *Chlamydomonas reinhardtii* cultivated under different light and temperature conditions. Panels **A**, **B**, the cells were grown in continuous light at 30 °C (**A**) or 39 °C (**B**); panels **C**–**F**, the cells were grown at 39 °C and then placed into the dark after 4, 8, 12, and 16 h and kept at 30 °C. Solid blue lines, full circles, kinase activity in cultures grown in continuous light at 30 °C; solid red lines, full circles, kinase activity in cultures grown in continuous light at 39 °C; solid blue lines, diamonds, kinase activity in cultures transferred from light at 39 °C to darkness at 30 °C. Solid blue lines, empty circles, DNA content in cells grown at 30 °C; solid red lines, empty circles, DNA content in cells grown at 39 °C; dashed blue lines, full circles, DNA content in cells transferred from 39 to 30 °C. Times of transfer into darkness are indicted by vertical lines and by dark boxes above the graphs.

Activities in whole cell lysates were followed in aliquots of synchronized populations grown in light at 30 or 39 °C, and subsequently transferred from 39 °C to darkness at 30 °C (Figure 7). At 30 °C, CDK activity peaked at two distinct time points (Figure 7A). A smaller, less pronounced peak correlated with the attainment of CPs (Figure 5A), and the larger, more pronounced peak correlated with the onset of DNA replication and preceded nuclear and cellular divisions (Figure 7A). Similar peaks were also observed at 39 °C. Their occurrence and duration were affected by temperature. The CP-related peak started at the same time as the control, but its duration was prolonged, in agreement with a slower attainment of CP at 39 °C (Figure 7B). The second, more pronounced peak was delayed at 39 °C; it preceded the onset of DNA replication and decreased thereafter (Figure 7B). Since no nuclear and cellular divisions occurred at 39 °C, the peak of whole cell lysate CDK activity seemed to be specifically related to DNA replication. Interestingly, the maxima of the two peaks were comparable at both 30 and 39 °C, although the extent of DNA replication differed between the two cultures (Figure 7A,B).

Transfer from light at 39 °C to darkness at 30 °C did not affect the timing of the CP-related peak, but in most cases, it resulted in a shift of timing and extent of CDK activity of the second peaks related to DNA replication, and possibly nuclear and cellular division. For transfer after cultivation for 4 h in light at 39 °C, the second peak occurred at a comparable time point as at 39 °C and preceded the onset of DNA replication. This was comparable to the peak in 39 °C grown cells with similar levels of DNA replication (Figure 7C). For cultures transferred to darkness at 30 °C after 8 h at 39 °C, the second peak of CDK activity occurred 3 h earlier (Figure 7D) and again preceded the onset of DNA replication, followed by nuclear and cellular divisions. The peak was more pronounced either because more DNA was replicated or because of the simultaneous occurrence of nuclear and cellular divisions. After transfer at 12 h, the increase in CDK activity started similarly to the 39 °C control, but reached the peak five hours later, and the CDK activity was double that of the peak at 39 °C (Figure 7E). The increase corresponded with the onset of DNA replication as well as nuclear and cellular divisions that occurred between 12 and 18 h. Once cell division was complete, CDK activity decreased. Transfer after 16 h at 39 °C occurred after the DNA replication-related peak was attained at 39 °C, and following the transfer, CDK activity increased again and attained another peak 2–3 h later. This peak corresponded to the completion of further DNA replication as well as nuclear and cellular divisions.

**Figure 8:**
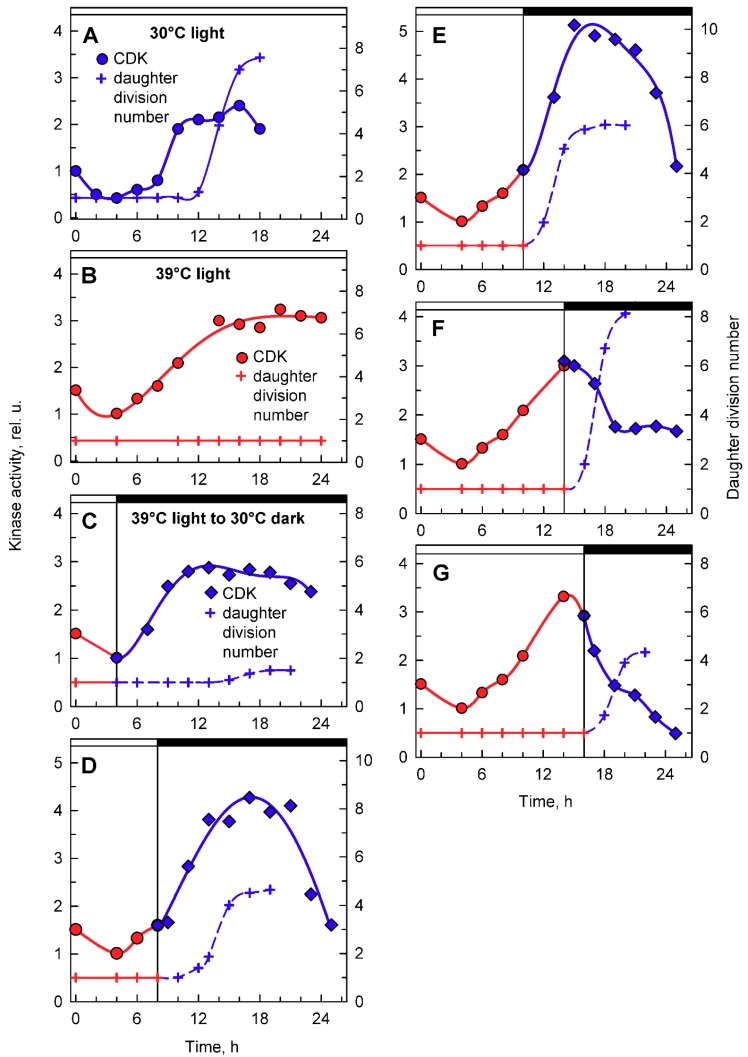
Activity of CDK in CrCKS1-bound fractions prepared from synchronized cultures of *Chlamydomonas reinhardtii* cultivated under different conditions of light and temperature. Panels **A**, **B**, the cells were grown in continuous light at 30 °C (**A**) or 39 °C (**B**); panels **C**–**G**, the cells were grown at 39 °C and then placed into darkness after 4, 8, 10, 14, and 16 h and kept at 30 °C. Blue lines, circles, kinase activity in cultures grown in continuous light at 30 °C; red lines, circles, kinase activity in cultures grown in continuous light at 39 °C; blue lines, diamonds, kinase activity in cultures transferred from light at 39 °C into darkness at 30 °C. Blue solid line, crosses, daughter division number—number of daughter cells released per mother cell at 30 °C; red solid line, crosses, daughter division number at 39 °C; blue dashed line, crosses, daughter division number after transfer from 39 °C to 30 °C. Times of transfer into dark are indicated by vertical lines and by dark boxes above the graphs.

To complement the analyses of whole cell lysate kinase activities, CDK activity bound to CrCKS1 beads was also assessed in the same set of experiments (Figure 8). At 30 °C, the CrCKS1 bead-bound CDK activity increased at the 10th hour and stayed elevated until the 18th hour, when it then decreased (Figure 8A). This peak corresponded with the onset of DNA replication (Figure 4B. blue curve), was maintained over three rounds of DNA replication and nuclear and cell divisions (Figure 2), and fell after they were completed. At 39 °C, the increase in CDK activity started about two hours earlier, reached similar levels as at 30 °C, and never decreased (Figure 8B), possibly because of the absence of nuclear and cellular divisions. In the culture transferred from 39 °C to darkness at 30 °C after 4 h in light, the increase in kinase activity occurred earlier compared to its mother culture at 39 °C, but the final activities were comparable. The increase in CDK activity preceded, by about 5 h, the onset of cell division in a small proportion of culture, and the activity started to decrease slightly once cell division was complete (Figure 8C). For cultures transferred to darkness at 30 °C after 8 or 10 h at 39 °C, CDK activity exceeded that of the original culture at 39 °C within 3 h of transfer, reached a peak at the completion of cell division, and decreased thereafter (Figure 8D,E). The culture grown at 30 °C for 14 or more hours completed cell divisions and released daughter cells (Figure 2, Figure 5A and Figure 8A). Nuclear and cell divisions were blocked at 39 °C, but when cultures grown at 39 °C were transferred into darkness at 30 °C, divisions occurred almost immediately. Within one hour of transfer, fifty percent or more of cells completed the first nuclear and cellular divisions (Figure 8F,G). Nuclear and cellular divisions were accompanied by decreased kinase activities (Figure 8F,G).

## 4. Discussion

Cultivation temperature affects the entire metabolism of cells, including their ability to complete the cell cycle. The sensitivities of different metabolic processes are not the same, as shown by the temperature optima for growth and photosynthesis, which can differ by as much as 10 °C [38]. Similarly, the upper temperature limits for growth, photosynthesis, and respiration can differ [38]. The ability of cells to divide seems to be the most temperature-sensitive process, as cell division is blocked even when growth is barely affected. Indeed, cell division of *C. reinhardtii* was blocked by cultivation at 39 °C, but growth was comparable between 30 and 39 °C (Figure 3, Figure 4, Figure 5 and Figure 6). Cells cultivated at 39 °C exhibited two main effects: (1) cell cycle arrest (Figure 3, Figure 5 and Figure 6) and (2) accumulation of starch (Figure 4); these are probably interconnected. Within the physiological range, a temperature increase of 10 °C generally leads to a speeding up of metabolism by about two-fold. This is also true for the duration of the cell cycle in *C. reinhardtii* [2] or in *C. eugametos* [3]. The fact that growth was not enhanced in the present experiments, as would have been expected within the physiological temperature range, suggested that the higher temperature had some inhibitory effects that lessened the growth rate, which was only slightly higher than at 30 °C (i.e., to about half of the predicted one). A similar decrease in growth rate, accompanied by successive prolongation of the cell cycle, as well as its individual steps, was shown in cultures of *C. reinhardtii* [2] and *C. eugametos* [3] cultivated at temperatures above the optimum. It is, thus, to be expected that a further increase in temperature up to 39 °C, as used in recent experiments, would cause a complete arrest of nuclear and cellular division. It has been established that yet another increase in temperature to a heat shock temperature of 42 °C has numerous effects on *C. reinhardtii*; it not only arrested the cell cycle but also caused significant inhibition of growth processes. There was only about a 1.6-fold increase in total protein within 24 h of heat shock treatment at 42 °C [35], compared to about a 10-fold increase in total protein within the same period at 39 °C (Figure 6B). In contrast, 39 °C treatment had only a limited specific effect; it negatively affected the cell cycle and positively affected starch accumulation. Cell growth at 39 °C was not affected for about 18 h and continued during the period of DNA replication and nuclear and cellular divisions in cultures grown at 30 °C. This led to the accumulation of giant nondividing cells rich in starch. Among the growth parameters, starch was outstanding as the only one showing a striking difference between cultures grown at 30 and 39 °C (Figure 4). In the control cultures, cells increased their starch content more than 10-fold (up to 80 pg cell^−1^, Figure 4), but a majority of this was used during cell division, even though the cells were grown in continuous light (Figure 4). This confirms that cell reproduction is an extreme energy-demanding process, in which large amounts of energy are required for DNA replication and nuclear and cellular divisions, as was previously shown [17,39]. Correspondingly, the blockage of cell division at 39 °C and prevention of starch expenditure led to the accumulation of high levels of starch. At 39 °C, this was further accentuated by faster starch production at high temperatures and high light (Figure 4). Over-accumulation of starch by a block in cell division has been a recurring theme, as it has been shown for *Chlorella vulgaris* starved of nitrogen, sulfur, and phosphate [25]; nitrogen- or phosphorus-starved *Desmodesmus quadricauda* [40,41]; as well as in nitrogen- and sulfur-starved *C. reinhardtii* [42,43]. Starch accumulation was also induced when nuclear DNA replication was inhibited [44] as well as by high temperature [45]. In contrast, the heat shock treatment of *C. reinhardtii* led to only a 46% increase in starch content over the 24 h period, and energy reserves were mostly accumulated in the form of lipids [35]. In algae capable of producing both starch and lipids, starch overproduction is the first response to stress, and with prolongation, it is replaced with lipid production [46,47] possibly to store more energy in a more compact form for stress recovery [48].

The cell cycle arrest at 39 °C was maintained even in darkness but was removed once the cells were shifted to 30 °C (Figure 5). With prolonged incubation at 39 °C, the ability of cells to fully recover from the block was decreased. The first affected process was daughter cell splitting; the second, the extent of cell division and the number of daughter cells formed; and the third, the extent of DNA replication (Figure 5 and Figure 6). A similar order of sensitivity, with cell division being the most sensitive process, was also shown for cell cycle arrest following exposure to different concentrations of cadmium in *Desmodesmus quadricauda,* a green alga that does not split daughter cells [49]. Likewise, the formation of palmelloids (i.e., unsplit daughter cells) was characteristic for the salt stress response [50]. Thus, the response to high temperature seems to follow general rules or mechanisms involved in stress responses. The incomplete recovery from cultivation at 39 °C is in conflict with the presumably complete recovery from heat shock at 42 °C [35,51]. This might seem surprising because the heat shock conditions of 42 °C were more severe than cultivation at 39 °C. However, the heat shock experiments were carried out with asynchronous cultures, where only about 20% of the cells in the population were dividing at 25 °C, which is the cultivation temperature used in the experiments [35]. Subtle changes seen in the synchronized cultures grown at 39 °C would therefore be impossible to detect.

The cell cycle arrest at 39 °C was the most striking effect of supraoptimal temperature. Its occurrence under heat shock conditions of 42 °C was hypothesized to be caused by reduced CDK activity [35], but it has never been tested; thus, the mechanism has remained enigmatic. In principle, there are two possible mechanisms: (1) cell cycle arrest due to low CDK activity and/or (2) a cell cycle block downstream of CDK activity. Of all the reproductive processes, DNA replication was the least sensitive to high-temperature treatment (Figure 4 and Figure 6). Similarly, DNA replication was not affected by high-temperature treatment of *Chlorella vulgaris,* and, instead, the block was just prior to nuclear division [6]. In fact, in *Chlorella vulgaris*, several rounds of DNA replication were performed, as the final DNA content was 16-fold higher than initial values [6]. In *C. reinhardtii*, DNA replication increased between two- and four-fold (Figure 4 and Figure 6), so DNA replication was not only delayed in timing but occurred to a lesser extent even at a supraoptimal temperature. Kinase activity in whole-cell extracts preceded DNA replication, decreased once DNA replication ceased, and, thus, apart from timing, did not differ between 30 and 39 °C (Figure 7). In a simplified system of fission yeast, it was shown that low levels of CDK activity allows DNA replication to occur, whilst higher levels are required for nuclear division [52]. This would fit the hypothesis of reduced CDK activity, allowing DNA replication to occur but being unable to switch on nuclear division. Notwithstanding the fact that the *C. reinhardtii* cell cycle is regulated by a more complex network of two CDKs, this hypothesis does not fit the data on mitotic kinase activities (Figure 8). Instead, mitotic kinase activities were comparable between 30 and 39 °C. The main difference between the two treatments was stabilization of a high mitotic CDK activity that only decreased after nuclear and cellular divisions (Figure 8). This supports the idea that the cell cycle block occurred downstream of CDK. The mitotic CDK behavior seems to phenocopy that of CDKA kinase in the anaphase-promoting complex (APC) mutant, *cdc27-6*, with persistent slightly higher kinase activity compared with control cells [21]. This implies involvement of APC in the supraoptimal temperature-induced cell cycle arrest. APC, the main E3 ubiquitin ligase to control mitotic progression and exit in all eukaryotes [53], has been shown to inhibit both CDKA and CDKB in *C. reinhardtii* [21]. In opisthokonts, APC is activated by mitotic kinases but kept inactive by spindle assembly checkpoint until all chromosomes are properly assembled [54]. Tubulin polymerization is known to be temperature sensitive [55]. Thus, it could be assumed that microtubule and, hence, spindle stability would be affected by supraoptimal temperature, leading to activation of the spindle assembly checkpoint, inhibition of APC, and maintenance of mitotic kinase activity. In the present experiments, the completion of cell division lagged behind the increase in mitotic kinase activity. The delay was successively shortened with the time the culture spent at 39 °C (Figure 8, compare A and D to E, F, G). This suggests that many of the processes required for completion of mitotic and cellular division may run even at 39 °C, and the cell cycle arrest is located very shortly before the onset of CDK inactivation. This further supports the idea of microtubule stability and possibility of the spindle checkpoint being involved in the arrest.
